# Mental health, resilience, and culturally congruent care among Muslim and Arab American women in the United States: a scoping review

**DOI:** 10.3389/fgwh.2026.1931567

**Published:** 2026-10-16

**Authors:** Lina Mohammad

**Affiliations:** West Coast University - Ontario Campus, Ontario, United States

**Keywords:** acculturation, Arab American women, culturally congruent care, Islamophobia, mental health, Muslim American women

## Abstract

The purpose of this scoping review was to explore the evidence regarding mental health outcomes, wellbeing status, help-seeking patterns, and culturally congruent care for Muslim American, Arab American, and Arab Muslim American women within the U.S. healthcare system. The review was guided by the Arksey and O'Malley framework and reported according to the Preferred Reporting Items for Systematic Reviews and Meta-Analyses extension for Scoping Reviews (PRISMA-ScR) checklist. The search was guided by the Population, Concept, and Context (PCC) framework. Articles were sought from PubMed Central, CINAHL, and Scopus, targeting peer-reviewed articles published between 2016 and 2026. Data were analyzed through narrative synthesis. The review included 29 studies, and five overarching themes emerged: (1) high mental health burden across multiple domains; (2) multi-level barriers to help-seeking and care access; (3) discrimination, Islamophobia, and provider bias as structural stressors; (4) mental health vulnerabilities and unmet care needs during the perinatal period; and (5) resilience, faith, and culturally congruent care as protective factors. Mosque-based, culturally congruent, faith-integrated interventions have demonstrated good acceptability, although evidence of their effectiveness remains preliminary. These findings highlight the need for greater investment in cultural humility training for providers, disaggregated national data on mental health, and intersectional longitudinal studies to improve equitable mental health care for these populations.

## Introduction

The number of Muslim and Arab American women continues to grow in the US, yet these women reportedly remain underserved in the American healthcare system (1). The 2020 US Religion Census reported that there were about 4.5 million Muslims and over 3.5 million Arab Americans in the US (2, 3); notably, most Arab Americans are Christian, while Muslim Americans span a wide range of ethnic and racial backgrounds (2). Women constitute about half of the total number, and are exposed to a set of vulnerabilities based on gender, religion, race, and immigration status, which together contribute to their mental health burden and access to care (1, 4). For instance, Kakoti (4) noted that these women face anti-Arab and anti-Muslim discrimination resulting in psychological distress. These women disaggregate to three related but distinct populations as stated throughout this review. First, Muslim American women refer to women living in America, who identify with the Islamic faith, regardless of ethnic/racial background. The second category is Arab American women, who encompass those of Arab ethnic origin but live in the US, who may hold any or no religious affiliation. Lastly, the Arab Muslim American women refer to the subgroup at the intersection of both identities, residing in the US.

Due to the potential sociocultural challenges and inequalities, studies have consistently reported that Arab American and Muslim American women have high rates of depression, anxiety, post-traumatic stress disorder (PTSD), and other forms of psychological distress (5–9). Coupled with post-migration trauma, intimate partner violence, these women significantly suffer from Islamophobia, a discrimination based on the Islamic faith (6). For instance, Samari et al. (10) explicitly linked Islamophobia with poor mental health outcomes, such as depression and anxiety. Further evidence has also linked the hijab identity with discrimination in the healthcare settings (11, 12).

Alongside the documented inequalities, these populations – Muslim American, Arab American, and Arab Muslim American women – reportedly remain underserved in the US mental healthcare systems (1, 4). Their poor access to mental healthcare services has been linked with many sociocultural barriers, including stigma, cultural mistrust of providers, preferences for gender-concordant care, modesty concerns, language differences, and provider unfamiliarity with Islamic beliefs (1, 13, 14). Unfortunately, studies have noted that Muslim and Arab American women who seek the mental care services sometimes encounter culturally-incompetent services, which only worsen the disparity and overall health outcomes (15, 16).

Some scholars have consequently proposed the application and reinforcement of culturally congruent care to counter the existing disparities and to ensure equitable service delivery (17). Unfortunately, there is only fragmented pieces of evidence regarding the culturally congruent care interventions for the Muslim and Arab American women in the US, with no scoping review ever done to explore the evidence. Therefore, this review addresses this gap by exploring the current evidence regarding mental health outcomes, help-seeking behaviors, barriers to help-seeking and mental healthcare, protective factors (including faith and resilience), and culturally congruent approaches to mental healthcare for Muslim American, Arab American and Arab Muslim American women in the United States from 2016 to 2026.

## Methods

### Study design

A scoping review approach was applied based on the Arksey and O'Malley (18) framework for scoping reviews [refined by Levac, Colquhoun, and O'Brien (19)]. Reporting was done according to the Preferred Reporting Items for Systematic Reviews and Meta-Analyses extension for Scoping Reviews (PRISMA-ScR) checklist (20). Scoping review is an appropriate approach for seeking evidence from heterogeneous study designs, and it would also help to spot the knowledge and research gaps for subsequent investigations.

### Research question

This review sought answers to one principal research question: what is the status of mental health, help-seeking behaviors, and culturally congruent care among Muslim and Arab American women in U.S. healthcare settings? This question focuses on three overarching elements in this population; the mental health status, barriers and facilitators (resilience), and the culturally congruent healthcare services provision in the U.S. healthcare contexts. Therefore, the research question aligns with the Population, Concept, and Context (PCC) framework recommended by the Joanna Briggs Institute (21).

### Inclusion and exclusion criteria

The inclusion and exclusion considerations are summarized in Table 1.

**Table 1:** Inclusion and exclusion criteria.

Criterion	Inclusion	Exclusion
Population	Adult women (≥18 years) identifying as Muslim American, Arab American, and/or Arab Muslim American	Studies exclusively involving men, children and adolescents;
Concept	Studies about mental health outcomes, wellbeing, psychological distress, help-seeking, culturally congruent/competent care.	Studies not related to mental health
Context	United States healthcare settings	Studies conducted exclusively outside the U.S. and with not data from the US healthcare settings.
Study design	Peer-reviewed empirical studies (quantitative, qualitative, mixed-methods), systematic reviews, scoping reviews, narrative reviews, and studies about scale validation.	Conference abstracts, dissertations, grey literature, book chapters, editorials, web essays, and non-peer-reviewed publications.
Language	English	Non-English publications
Date	January 1, 2016 - June 16, 2026	Before January 1, 2016
Access	Full text accessible.	Only the abstract accessible.

### Search strategy

Articles were sought from three main online journal databases, including PubMed Central, CINAHL and Scopus. Additional search was done on Google Scholar, Google manual search and the reference lists of the identified articles. The search strings used both MeSH terms and free-text keywords according to the PCC considerations (Supplementary Appendix A). The keywords of focus include Muslim and Arab American women; mental health and wellbeing; psychological distress; help-seeking behaviors; culturally congruent care; United States healthcare settings. The search was filtered on the databases following the inclusion and exclusion criteria in Table 1.

### Study selection and data extraction

All the records were retrieved and tallied in a reference management system (Mendeley), and screening was done in two phases. The first phase focused on the title and abstract while the second phase, which was more refined, examined the full text. Screening was done by the lead author and two additional trained reviewers (paid research assistants) according to the set eligibility criteria. Cases of disagreements were resolved by a third reviewer. Inter-rater reliability was considered, and were all within the acceptable score (*κ* ≥ 0.79), hence accepted (22).

The entire process of article selection was summarized in the PRISMA-ScR flow diagram (Figure 1). Accordingly, the initial search for the relevant articled resulted 1,031 articles from the databases and 107 from the manual searches. Therefore, the initial search gave a total of 1,138 articles. However, 261 articles were removed as duplicate copies, leaving 877 unique entries. The quick assessment of the titles and abstract also resulted in elimination of 493 articles as irrelevant, leaving 384 articles for screening. A total of 196 articles were not retrieved, leaving 188 articles. Additional exclusion was done based on three criteria: non-peer review (*n* = 95); studies done outside the US healthcare context (*n* = 41) and studies not including women (*n* = 23). In the end, 29 articles met the inclusion criteria.

**Figure 1:**
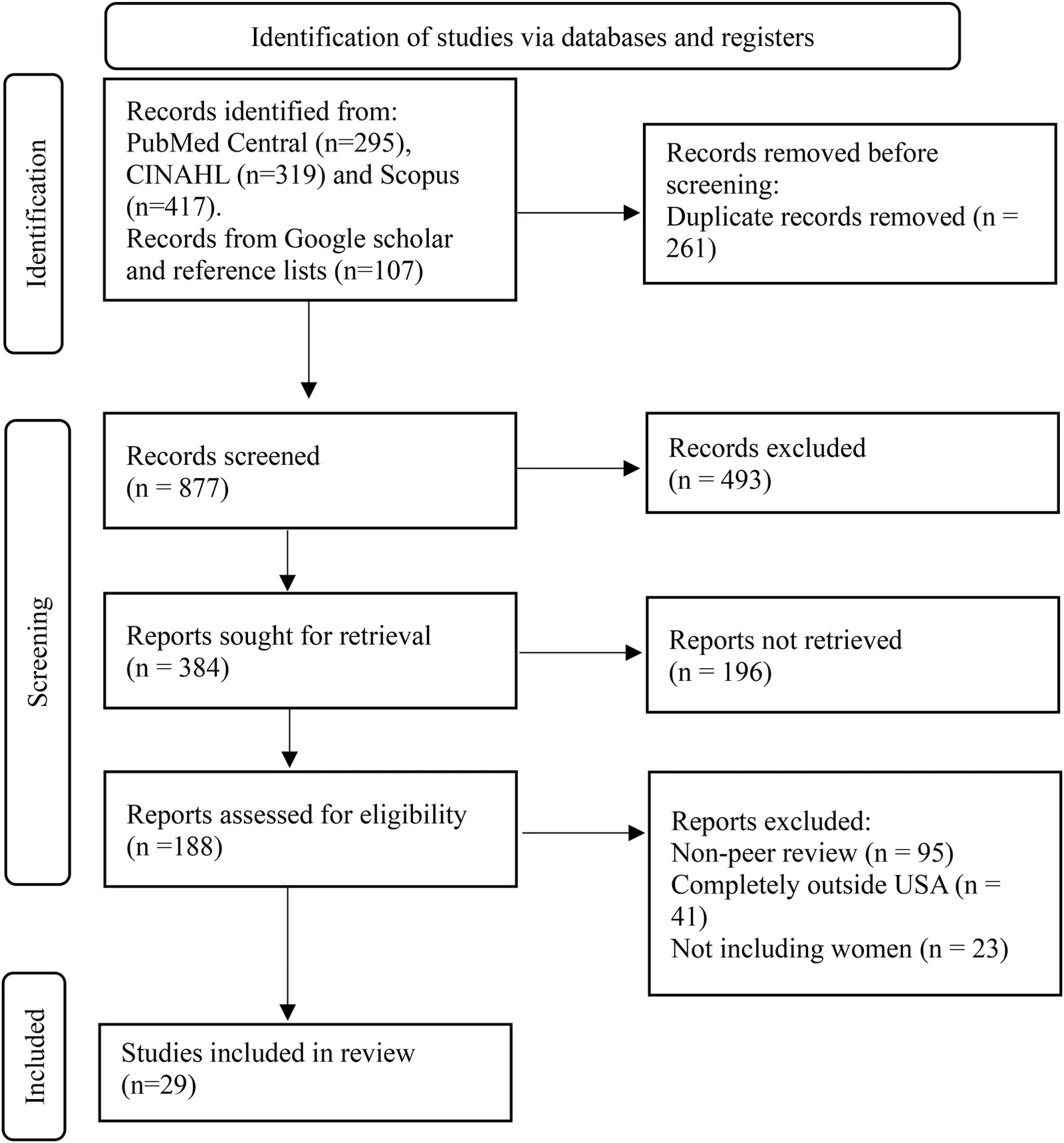
The PRISMA flow diagram.

The extraction of data was done using a standardized form was designed and pretested on five studies before being used to extract specific data from the articles: author(s) and year of publication, study design, sample/population, U.S. setting, mental health concept(s), key findings, culturally congruent care elements, barriers identified, and protective/resilience factors (Supplementary Appendix B). It is important to note that since this review included both primary and secondary research, all the studies with secondary data, such as scoping and systematic reviews, were manually screened, checking their data tables against the current primary studies to avoid duplication. Additionally, the reference lists of the included reviews (5, 7, 16, 24–28), were also examined carefully to check any potential duplication. This process ensured no duplication in this review, and all the reviews included in this paper were all independent data sources.

### Data synthesis

A descriptive narrative synthesis protocol was used as outlined in Lucas et al. (23); that involves reading the papers to identify related or similar concepts, which are then grouped, and used to generate the overarching themes according to the study aim. The analysis highlighted five thematic areas: (1) Mental health outcomes and prevalence; (2) Barriers to help-seeking and care uptake; (3) Resilience, faith and protective factors; (4) Culturally congruent help-seeking and care models and interventions; (5) Research gaps and implications for investments in women's mental health. All included studies are compared in a summary evidence table by domain (Supplementary Appendix B).

## Results

### Study selection

The search yielded a total of 29 articles that met the inclusion criteria. However, it should be noted that despite the strict focus on the US and women gender, this review also included three articles that had mixed-gender and multinational data since they provided contextually relevant evidence that was considered crucial in this review, and not present in the U.S. women-specific literature (Supplementary Appendix B). The 29 articles were published between 2016 and 2025 that focused on different thematic concepts around the study aim, including mental health outcomes and prevalence; barriers to help-seeking and care utilization; discrimination, Islamophobia and psychological distress; perinatal and maternal mental health; culturally congruent care, and resilience and faith-based protective factors.

### Characteristics of included studies

#### Study designs and methodologies

The included studies employed diverse methods and designs. There were eight reviews (5, 7, 16, 24–28). The were twelve quantitative cross-sectional or survey-based studies [*n* = 12 (1, 8, 11–14, 29–34)]. Four studies applied qualitative methods (35–38). Two studies used the mixed methods (17, 39). There were also single studies on scale validation study (34), curriculum development (40), and one cross-sectional survey examining perinatal bereavement (41). All the eight secondary sources were cross-checked against the 21 primary studies to ensure no overlap. The studies' sample sizes varied, ranging from eleven (37) to 1,214 (10).

In terms of geographic distribution and settings, most studies were conducted in the Midwest and Great Lakes region of the United States. There was a notably high number of studies conducted in Michigan (Detroit-Dearborn Arab American community corridor), Chicago, and Illinois, where the study participants were mostly sought from mosques and community care settings. Other regions also included San Francisco Bay Area (17), Arizona (36), Alabama (8), as well as nationwide (1, 32). The healthcare settings were relatively diverse, including hospital-based perinatal units, outpatient community clinics, community clinics at mosques, federally qualified health centers, and community online platforms.

Population characteristics varied significantly. Fifteen studies exclusively included Muslim American women as their participants, seven studies included Arab American women or women who speak Arabic as their primary language (categorized as immigrants or refugees), and four studies included both identities, or made overlapping community samples. Immigration and refugee status emerged as a significant feature, and most of the perinatal and trauma research focused on women from the first generation. Three studies (11, 37, 38) specifically explored the gender dimension in combination with ethnic and religious affiliations, and in one instance sexual minority.

### Thematic findings

The narrative synthesis identified five overarching themes, including (1) high mental health burden across multiple domains, (2) multi-level barriers to help-seeking and care access, (3) discrimination, Islamophobia, and provider bias as structural stressors; (4) mental health vulnerabilities and unmet care needs during the perinatal period; and (5) resilience, faith, and culturally congruent care as protective factors.

#### Theme 1: high mental health burden across multiple domains

Across the reviewed studies, there was a consistent finding of high rates of depression, anxiety, psychological distress, PTSD, and suicidal ideation in Muslim and/or Arab American women. For instance, in a scoping review of 23 studies, Almutairi et al. (7) found that mental health disorders were consistently reported at high prevalence, and that women from the Middle East who immigrated to the United States experienced low rates of mental health services usage, where acculturative stress, discrimination, and language barriers were found to be significant contributing factors. The most commonly studied outcome was depression, which was included in 18 out of the 29 studies. Additionally, Abuelezam and Fontenot (28) also reported high depression rates among Arab American women and linked it with immigration stress, intimate partner violence, social isolation, and acculturation conflict, and found that there were no culturally adapted depression screening tools in clinical settings within the United States.

One of the most noteworthy mental health outcomes is from a nationally representative analysis of the 2019 survey from the Institute for Social Policy and Understanding (32), which revealed that American Muslims were twice as likely as all other religious groups to report having attempted suicide at least once in their lifetimes (32). The findings from Awaad et al. (32) further reported that about 8% of Muslims reported a lifetime suicide attempt, compared to citing 6% of Catholics, 5% of Protestants, and 3.6% of Jewish respondents. Notably, the level of religiosity did not reduce this risk, contradicting the popular notion in Muslim communities that religiousness is a protective factor against suicidality. Awaad et al. (32) also reported that religious discrimination and community-level mental health stigma to be the greatest contributors to this health disparity, hindering help-seeking and access to care.

The compounded trauma burden in severely traumatized Arab immigrant women in the United States was also documented by Norris and Aroian (9), who showed that pre-migration persecution was a significant predictor of PTSD, while post-migration stressors, such as discrimination and acculturative stress were significant predictors of both PTSD and major depression disorder. In this study, social support was identified as the most salient post-migration protective resource. Notably, studies that contribute to this theme apparently drew on different populations: Almutairi et al. (7) and Norris and Aroian (9) sampled focused on Middle Eastern or Arab immigrant women without Muslim religious identity, whereas the findings from Awaad et al. (32) were drawn from a mixed-gender sample of Muslim Americans of varied ethnic backgrounds. Only Abuelezam and Fontenot (28) focused strictly on Arab American and Arab immigrant women's depressive conditions. Therefore, the “high burden” in this theme can be best understood as consistent across overlapping populations.

#### Theme 2: multi-level barriers to help-seeking and care access

The barriers to using mental health services and to help-seeking were the most common and well documented theme in the literature, ranging from individual, family, community to structural levels.

Internalized stigma was the most apparent barrier at the individual level. Internalized stigma as measured by increased vigilance was found to be significantly associated with depression (OR = 0.924, 95% CI = 0.888–0.962, *p* < 0.001) from the analysis of 373 Muslim women's responses across the United States (8). The authors explained a growing trend of “double jeopardy”: being discriminated against due to Muslim identity and mental illness at the same time, which further reduces access to health care.

At the family and community level, Alhomaizi et al. (33) used a socio-ecological approach in exploring the barriers to formal help-seeking among Arab-Muslims in the United States, and found that shame, concerns about family honor, and community pressure were the primary suppressors of formal help-seeking, especially among women. The stigma of psychiatric diagnosis and feelings of shame in the community for mental health problems reinforced the expectation that mental health issues were private matters of the family.

The structural barriers were well documented by Ali et al. (13) in a quantitative investigation. The study revealed that underutilization of mental health services was significantly predicted by a combination of negative beliefs about mental health causation, rejection attitudes toward formal services, and low familiarity with professional mental health services. Interestingly, the patterns significantly varied according to ethnicity, with Arab Muslim women showing lower familiarity with mental health services than White Muslim women, suggesting that ethnicity influences access pathways even within the Muslim community.

McLaughlin et al. (39) also found, in their mixed-methods analysis, that self-identified Islamophobia was positively linked to self-stigma of seeking help, which was further linked to negative attitudes towards help-seeking. Particularly, Muslim women more likely to self-identify with Islamophobia than the men. Participants strongly preferred same-religion or culturally competent therapists, which suggests that there are direct implications for workforce diversity in U.S. mental health settings. On the same idea, Baltimore and Kim (27) reiterated that these factors are structural barriers for middle eastern migrant women and that social workers serve as a missed opportunity to connect with care in this population.

These thematic findings predominantly come from Muslim-identified women samples (8, 13, 39), even though the study of McLaughlin et al. (39) included male participants. Alhomaizi et al. (33) is the clearest study exception that specifically studied Arab-Muslim individuals at the intersection of both identities. Interestingly, Ali et al. (13) further found that Arab Muslim women reported comparatively low levels of awareness regarding mental health services compared to the White Muslim women counterparts. This observation suggests that ethnicity may play a role in help-seeking barriers even within a shared Muslim identity.

#### Theme 3: discrimination, Islamophobia, and provider bias as structural stressors

A number of studies have reported the effects of discrimination, both in the community and in the U.S. health care system, regarding the mental health and care-seeking among Muslim and Arab American women. For instance, a survey of 254 Muslim women in Chicago conducted by Murrar et al. (12) revealed that the providers did not listen to them (29.8%), 25.4% of these respondents expressed being treated with less courtesy than a non-Muslim patient, and 24.3% felt that a provider treated them with less competency. These rates of perceived clinical discrimination are startlingly specific, and are a direct indictment of provider bias in U.S. healthcare settings. In this study, higher self-rated religiosity was seen to be negatively associated with perceived discrimination, which was an unexpected finding the authors felt could have arisen from the buffering effects through religious community support.

Hashem and Awad (11) developed a concept of 'solo status' (being the only Muslim in a certain context) as an important moderator of the psychological effects of discrimination. Among the 123 sample of Muslim women in the United States, it was reported that being the only Muslim in a care setting was a moderating effect between discrimination and psychological distress, especially for Muslim women who consistently wore the hijab. Consensual qualitative study by Abdel-Salam et al. (37) involving 11 Arab American women yielded a similar finding, that the psychological burden was overwhelming and that experiences of discrimination based on both ethnicity and gender (such as stereotyping, marginalization, and dual identities between Arab and American cultural norms) were key factors in this.

Findings on discrimination and provider bias in this theme are more focused on the Muslim-identity subgroup than then Arab ethnicity—this can be drawn from specific studies (11, 12). These studies sampled Muslim women without an Arab-ethnicity criterion, and hijab status, a religious marker, was the key moderating variable in Hashem and Awads' concept of solo-status findings (11). However, there is also an exception, where the researcher specifically focused on Arab ethnicity (37). Hence, the ethnicity-based discrimination did not receive comprehensive examination as compared to Arab religion.

#### Theme 4: mental health vulnerabilities and unmet care needs during the perinatal period

The sub-theme of perinatal mental health was unique and significant, comprising seven studies, which included postpartum depression, antenatal anxiety, maternal bonding, maternal bereavement, and maternal healthcare among Muslim and Arab American women.

In a cross-sectional study of 115 Arab immigrant women living in Dearborn, Michigan, Alhasanat-Khalil et al. (29) determined that economic vulnerability, antenatal anxiety, higher education, and lower social support significantly predicted PPD symptoms. The study further revealed that 88% of their participants lived in households earning less than $40,000 annually and 68% preferred being interviewed in Arabic, which point at the confounding effects of economic and language in care-seeking. In their follow-up study in 2019, Alhasanat-Khalil and colleagues (30) revealed that 25% (*n* = 29/115) of the women met the clinical cut-off score of 10 or higher on the Edinburgh Postnatal Depression Scale, and that the level of PPD symptoms was positively associated with marginalization. Moreover, Khalil et al. (31) found that acculturative stress, trauma exposure and depressive symptoms were all significant predictors of impaired maternal bonding among immigrant and refugee Arab American women in Detroit.

Another qualitative study involving Muslim refugee women in Arizona found that language barriers, immigration-related fears, provider cultural humility, and the absence of professional interpreters were the primary barriers to accessing maternal healthcare services (36). In addition, Khalil et al. (41) reported that the Arab Muslim women who had experienced perinatal loss in the U.S. hospitals disappointedly met providers who were not knowledgeable about Islamic bereavement practices, including ritual washing, prayer, and timing for burial. Additionally, the scoping review by Simonovich et al. (24) found that prayers and other religious beliefs, such as reciting parts of the Quran, saying a Dua, and listening to audio recordings of prayers are effective in reducing anxiety, depression, stress, pain and fear among Muslim women during pregnancy, childbirth, unexpected cesarean section and moments of infant loss. The authors subsequently recommend incorporating prayers and other faith practices into U.S. perinatal clinical settings as non-pharmacological methods. Important to note, unlike Themes 2 and 3, the perinatal evidence base draws primarily on Arab ethnicity rather than Muslim religious identity (29–31). Only Khalil et al. (41) centered on the Arab Muslim intersection explicitly under this theme.

#### Theme 5: resilience, faith, and culturally congruent care as protective factors

Despite the significant mental health burden, there is evidence supporting faith, community, and culturally congruent care as protective and acceptable among this population. The study by Simonovich et al. (24) found that incorporating faith practices helps to reduce the perinatal depression and anxiety among Muslim women, recommending its application in clinical settings for non-pharmacological management of perinatal mood disorders. This finding has clinical implication; that enlightening antenatal and postpartum women about the significance of their faith-based practices may be useful in helping to alleviate perinatal mood symptoms can help establish trust with the care providers.

In a qualitative study of mosque-based intervention design preferences involving 19 Muslim women in Chicago, Vu et al. (35) uncovered a strong preference among women for interventions that were integrated with their faith, female-only, and delivered within the mosques. The respondents further expressed that inclusion of Islamic values and trusted community messengers is key to acceptable and effective care delivery. On the same note, Awaad et al. (17) showed that this model can be applied in the San Francisco Bay Area, where a family-centered, faith-integrated, psychoeducation model for mental health literacy was shown to decrease mental health stigma and improve mental health knowledge among Muslim participants in a community-based participatory partnership with a mosque.

Moreover, Saherwala et al. (16) demonstrated a framework for culturally competent mental health care for Muslim women in the U.S., which stress the need to recognize Islamic beliefs without diminishing religious coping. The framework also advocates for reducing medication stigma, maintaining modesty in mental health spaces, and arranging for female providers to attend to female patients in a clinically appropriate way. Still on the same issue, Vu et al. (14) reported that 53% of Muslim women in their survey, in Chicago, indicated that they delayed to seek care since female providers were not available. The same study by Vu and colleagues (14) indicated that higher religiosity (OR = 5.2, *p* < 0.01) and modesty (OR = 1.4, *p* < 0.001) were independently associated with delay in seeking care. Importantly, being in the United States for more than 20 years was associated with a lower risk of care delay (OR = 0.22, *p* < 0.05), indicating that acculturation and familiarity with the U.S. health care system over time diminishes—but does not eliminate—the burden of care barriers related to religion.

Additionally, Awaad et al. (34) developed a validated measurement tool specifically for Muslims, called the Muslims' Perceptions and Attitudes to Mental Health (M-PAMH) scale, which was tested among the American Muslims. Accordingly, the three most important factors predicting mental health service underutilization were negative beliefs about mental health, rejection attitudes toward mental health services, and low familiarity with professional care.

Finally, the accumulated stressors coming from ethnic, religious, and sexual minorities had a compounded effect on mental health, and affirming providers who could hold all three were exceedingly rare, in U.S. settings, as described by Chaudhry et al. (38) in their interviews conducted among 20 Arab sexual minority women migrants to the United States. Addressing this provider gap directly, Rashed et al. (40) implemented a medical school curriculum intervention that integrated content on Arab and Muslim health into formal medical school education. The intervention included some mental health stigma, gender and religious practices, and found an improvement in provider knowledge, as measured by pre-post assessment.

Under this theme, nearly all the studies focused on religious practice as the protective mechanism (14, 16, 24, 34, 35). However, Awaad and colleagues did not strictly include women (17). Again, Chaudhry et al. (38) is another partial exception, that sampled Arab sexual minority women migrants, where ethnic, religious, and sexual-minority identities compounded together rather than religion acting alone. This theme thus leans more towards faith-based protective factors than Arab ethnicity.

## Discussion

This review noted a disproportionate, yet significantly underrecognized mental health burden among the Muslim and Arab American women, created not only through individual vulnerability, but also by the interaction of structural, sociocultural, and systemic processes. Unfortunately, the burden still falls of appropriate care in the current U.S. healthcare system due to insufficient services that fail to meet the needs of culturally congruent and equitable care for this population.

The mental health status of this population is notable as deeply significant concept that encompasses diverse conditions. Depression, anxiety, PTSD, perinatal distress, and suicidality were all significantly higher in many studies, including the study by Awaad et al. (32), which revealed that American Muslims have double the lifetime risk of suicide attempts than all other religious groups. This observation directly challenges the notion that faith alone is protective against suicidality. These findings reflect some of the tenets of Meyer's minority stress theory (42), which suggests that chronic and cumulative stressors, such as discrimination, stigma, and prejudice can create an added psychological burden for minorities communities more than the majority population.

The minority stress framework is especially relevant to Muslim and Arab American women, who hold multiple minority statuses, such as gender, religion, ethnicity, and immigration status. Backed by foundational work by Crenshaw (43, 44), this work has shown that overlapping social identities are not additive but rather multiplicative as they create patterns of disadvantage that are simultaneously unique and compounding, and that cannot be understood by only examining one axis of identity at a time. Additionally, the findings of this review shed light on the lived realities of intersectionality for Arab sexual minority women, gendered ethnic expectations, and hijab-related solo status as experienced by these women in clinical and structural settings.

Certain elements of social and structural discrimination within U.S. health care institutions were identified as direct triggers of mental health disparities. For instance, the systematic review of Samari et al. (10), involving 53 studies of Muslim adults found that Islamophobia was consistently linked to poor mental health, suboptimal health behaviors, and poor healthcare-seeking. Structural racism embedded in U.S. health systems, including anti-immigration policies, labor exploitation and culturally insensitive services, represents a structural barrier to accessing mental health services for immigrant populations. This form of barrier is reflected in the framework applicable to Muslim and Arab American women whose immigration status, religious identity and ethnic minority status are intertwined with systemically inequitable care structures (45). Moreover, the findings of Murrar et al. (12), who found that almost one-third of Muslim women indicated that providers did not listen to their needs, and the findings of Vu et al. (14), who found that 53% delayed care cases arose from absence of female provider squarely situates the problem at the institutional level and within the providers' behaviors/competencies.

The patterns of barriers to help-seeking presented herein reflect some previous observations found in the wider immigrant mental health literature, including cases of internalized stigma, family and community honor-based suppression of disclosure, and discrimination-induced self-stigma (8, 33, 39). In their study of Black immigrant women, Pederson et al. (46) also identified a multitude of social determinants that converged to create mental health disparities and reduced engagement in mental health treatment, a process similar to what this review found for Muslim and Arab American women. Low mental health literacy has been reported as a structural barrier, which many researchers have pointed out as requiring additional psychoeducation and community outreach programs to properly invest in the mental health of underserved populations (27, 47).

Perinatal mental health is one of the most clinically pressing areas of this review. The evidence garnered from many researchers on predictors of postpartum depression, impaired maternal bonding, barriers to maternal care access, and Islamic bereavement needs, all point to a critical window of vulnerability that has largely been ignored by U.S. maternal health systems (29, 30, 36, 41). This is a critical concern that can spur the negative effects of the untreated PMD that can extend beyond the individual woman to children's cognitive, emotional and developmental outcomes, both in the present and in the future (24). The absence of knowledge of Islamic bereavement practices in U.S. perinatal is another example of how health care institutions have not fully met the needs of this population, not by their malicious intent, but by their structural omission (41). As such, the healthcare system has failed to provide the knowledge and training to service providers, and their policies did not reflect the existence of Islamic bereavement practices.

The review's most positive findings and the most clear-cut call to invest in culturally congruent care are found in the evidence on faith, resilience and culturally congruent care. Evidence from various religious communities has emerged that spirituality and religiosity constitute powerful protective factors for mental health. Specific evidence on Islamic religiosity has linked religiosity with lower depression and anxiety, better coping with traumatic stressors, and higher psychological resiliency (47). Due to this notable protective role, Simonovich et al. (24) suggested the need for using faith practices as non-pharmacological measures to treating mental health concerns in perinatal care settings. This is aligned with the recent literature consensus that the inclusion of patients' spiritual and cultural perspectives in care is not a trade-off for the rigor of care, but can be used as a complementary. This shift is rooted in the seminal work of Tervalon and Murray-García (48), which called for a lifelong practice of self-evaluation, critique, and redressing power imbalances when treating patients. Instead of the “competence” approach, which is inherent in most of the studies included, cultural humility requires that providers treat patients as experts in their own cultural, religious, and experiential reality (16, 35, 36).

One of the most promising structural interventions to the documented barriers is community-based participatory research (CBPR) models, such as the one already done by Awaad et al. (17) in partnership with the mosque. On the same point, De las Nueces et al. (49) conducted a systematic review that revealed that CBPR was especially effective at recruiting and retaining racial and ethnic minority participants and at producing significant intervention effects for these groups. More recently, a systematic review by Chen et al. (50) found that CBPR interventions resulted in meaningful improvements in mental wellbeing outcomes among ethnic minority, immigrant, and refugee populations. CBPR is a method that is applicable to Muslim and Arab American women, and can take the form of clinical research that also aims to build trust in the community; that utilizes community-based and community-situated social and religious institutions, like mosques; and that engages communities as active partners in their own mental health systems, not as passive beneficiaries of outsider services.

Another notable longstanding gap revealed by this review is the absence of Arab Americans in federal health data in the previous years. It was only in March 2024 when a major policy shift that took place, where the OMB's added a dedicated category for Middle Eastern and North African Americans (MENA) on federal forms, separately from the White racial category (51). As Abuelezam et al. (5) emphasized, this lack of classification has long made the mental health disparities of Arab American women statistically invisible. The operationalization of the MENA category in health surveillance systems, insurance claims and clinical registries will for the first time provide an epidemiological infrastructure to support and justify investment in mental health services targeted at this population, while providing the basis for population-level research into mental health disparities.

By considering these themes together, the review findings reveal an uneven evidence base across the three populations under study. Findings on discrimination, Islamophobia, and faith-based resilience are drawn predominantly from Muslim-identified samples, regardless of ethnicity. On the other hand, findings on perinatal mental health and maternal bonding are drawn predominantly from Arab-ethnicity samples, regardless of religious affiliation. Arab Muslim American women, who are at the intersection of both identities were the explicit focus of only a small number of included studies (33, 41), despite this group arguably facing the most compounded structural burden described throughout this review.

## Conclusion

This scoping review offers the most recent and most comprehensive synthesis of evidence about the mental health and wellbeing of Muslim and Arab American women in the U.S. healthcare sector between 2016 and 2026. The findings reflect that this has been burdened by high rates of mental health problems (depression, PTSD, perinatal distress, and increased suicide attempts) that arise from diverse factors, including Islamophobia, discrimination, structural insufficient care, and multilevel stigma. At the same time, the evidence points to the need for care models involving faith, community and culturally congruent care as strong as avenues for resilience and recovery. However, there is also a need to focus on action, such as training providers, redesigning care systems and investing in community-based, faith-integrated interventions that account for the identities and lived experience of an underserved and unrecognized community.

### Limitations

The studies included in this review may not be fully representative of the US healthcare system since it only focused on the English publications, and the absence of data from certain areas where research had not been conducted could limit generalizability. Also, it should be noted that three studies were included in this review but they used samples with mixed-gender and binational participants with some women-specific data not disaggregated, and this should be understood with caution. Finally, this review included secondary data sources whose level of evidence vary from the primary research studies.

## References

[1] Elahi S Elsayed D Ali S Awaad R. Ethnic differences in Muslim women’s mental health beliefs, rejection attitudes, and familiarity with professional mental healthcare. Ment Health Relig Cult. (2023) 26(9):890–907. 10.1080/13674676.2023.2243459 38585328 PMC10997350

[2] Arab American Institute. *National Arab American demographics*. (2024). Available online at: https://www.aaiusa.org/demographics (Accessed July 8, 2026).

[3] Grammich C Dollhopf E Gautier M Houseal R Jones DE Krindatch A. 2020 U.S. Religion Census: Religious Congregations & Membership Study. Lenexa, KS: Association of Statisticians of American Religious Bodies (2023). Available online at: https://www.usreligioncensus.org (Accessed July 8, 2026).

[4] Kakoti SA. Arab American women, mental health, and feminism. Affilia. (2012) 27(1):60–70. 10.1177/0886109912437572

[5] Abuelezam NN El-Sayed AM Galea S. The health of Arab Americans in the United States: an updated comprehensive literature review. Front Public Health. (2018) 6:262. 10.3389/fpubh.2018.00262 30255009 PMC6141804

[6] El-Refaay SM Kenny C Weiss S. Depression and anxiety among arab individuals in the United States: a meta-analysis. J Immigr Minor Health. (2025) 27(2):329–50. 10.1007/s10903-024-01648-9 39602001 PMC11903594

[7] Almutairi W Seven M Poudel-Tandukar K VanKim N. Mental health disorders among Middle Eastern immigrant women living in the United States: a scoping review. Perspect Psychiatr Care. (2022) 58(4):3079–102. 10.1111/ppc.13088 35419835

[8] Budhwani H Hearld KR. Muslim women’s experiences with stigma, abuse, and depression: results of a sample study conducted in the United States. J Womens Health. (2017) 26(5):435–41. 10.1089/jwh.2016.5886 28263695

[9] Norris AE Aroian KJ Nickerson DM. Pre-migration persecution, post-migration stressors and resources, and post-migration mental health: a study of severely traumatized U.S. Arab immigrant women. J Am Psychiatr Nurses Assoc. (2016) 17(4):283–96. 10.1177/1078390311408900 PMC4464810 21835819

[10] Samari G Helm HE Tabb KM. Islamophobia, health, and public health: a systematic literature review. Am J Public Health. (2018) 108(6):e1–9. 10.2105/AJPH.2018.304402 29672152 PMC5944883

[11] Hashem HM Awad GH. Hijab, solo status, discrimination, and distress among Muslim women in the U.S. Couns Psychol. (2024) 52(5):773–801. 10.1177/00110000241242808

[12] Murrar S Baqai B Padela AI. Predictors of perceived discrimination in medical settings among Muslim women in the USA. J Racial Ethn Health Disparities. (2024) 11(1):150–6. 10.1007/s40615-022-01506-0 36622571

[13] Ali S Elsayed D Elahi S Zia B Awaad R. Predicting rejection attitudes toward utilizing formal mental health services in Muslim women in the US: results from the Muslims’ perceptions and attitudes to mental health study. Int J Soc Psychiatry. (2022) 68(3):662–9. 10.1177/00207640211001084 33719665

[14] Vu M Azmat A Radejko T Padela AI. Predictors of delayed healthcare seeking among American Muslim women. J Womens Health. (2016) 25(6):586–93. 10.1089/jwh.2015.5517 PMC5912720 26890129

[15] Ahmed S Reddy LA. Understanding the mental health needs of American Muslims: recommendations and considerations for practice. J Multicult Couns Devel. (2007) 35(4):207–18. 10.1002/j.2161-1912.2007.tb00061.x

[16] Saherwala Z Bashir S Gainer D. Providing culturally competent mental health care for Muslim women. Innov Clin Neurosci. (2021) 18(4-6):33–9. Available online at: https://www.ncbi.nlm.nih.gov/pmc/articles/PMC8667712/ (Accessed July 8, 2026). 34980982 PMC8667712

[17] Awaad R Ali S Elsayed D Yusuf N Shaban-Nejad A Shim R. Addressing mental health through community partnerships in a Muslim community. Psychiatr Serv. (2022) 73(8):930–3. 10.1176/appi.ps.202100505 36065581

[18] Arksey H O’Malley L. Scoping studies: towards a methodological framework. Int J Soc Res Methodol. (2005) 8(1):19–32. 10.1080/1364557032000119616

[19] Levac D Colquhoun H Brien O K K. Scoping studies: advancing the methodology. Implement Sci. (2010) 5:69. 10.1186/1748-5908-5-69 20854677 PMC2954944

[20] Tricco AC Lillie E Zarin W O'Brien KK Colquhoun H Levac D. PRISMA Extension for scoping reviews (PRISMA-ScR): checklist and explanation. Ann Intern Med. (2018) 169(7):467–73. 10.7326/M18-0850 30178033

[21] Peters MDJ Marnie C Tricco AC Pollock D Munn Z Alexander L. Updated methodological guidance for the conduct of scoping reviews. JBI Evid Synth. (2020) 18(10):2119–26. 10.11124/JBIES-20-00167 33038124

[22] Cohen J. A coefficient of agreement for nominal scales. Educ Psychol Meas. (1960) 20(1):37–46. 10.1177/001316446002000104

[23] Lucas PJ Baird J Arai L Law C Roberts HM. Worked examples of alternative methods for the synthesis of qualitative and quantitative research in systematic reviews. BMC Med Res Methodol. (2007) 7:4. 10.1186/1471-2288-7-4 17224044 PMC1783856

[24] Simonovich SD Quad N Kanji Z Tabb KM. Faith practices reduce perinatal anxiety and depression in Muslim women: a mixed-methods scoping review. Front Psychiatry. (2022) 13:826769. 10.3389/fpsyt.2022.826769 35686180 PMC9170987

[25] Berry Y Khan S Smadi R Rehman S Hanania J Alabdalrazzak M. Mental health challenges and barriers to care among Arab refugees: a scoping review of US and Canadian studies. PRiMER. (2025) 9:37. 10.22454/PRiMER.2025.330739 40727864 PMC12303143

[26] Elshahat S Moffat T. Mental health triggers and protective factors among arabic-speaking immigrants and refugees in North America: a scoping review. J Immigr Minor Health. (2022) 24(2):489–505. 10.1007/s10903-021-01215-6 33987797

[27] Baltimore K Kim W. Mental health service use among Middle Eastern migrant women: social work’s role in promoting mental health literacy. Adv Soc Work. (2024) 23(2):205–219. 10.18060/27061

[28] Abuelezam NN Fontenot HB. Depression among Arab American and arab immigrant women in the United States. Nurs Womens Health. (2017) 21(5):395–9. 10.1016/j.nwh.2017.08.003 28987212

[29] Alhasanat-Khalil D Fry-McComish J Dayton C Benkert R Yarandi H Giurgescu C. Acculturative stress and lack of social support predict postpartum depression among U.S. Immigrant women of arabic descent. Arch Psychiatr Nurs. (2018) 32(4):530–5. 10.1016/j.apnu.2018.02.005 30029744

[30] Alhasanat-Khalil D Giurgescu C Benkert R Fry-McComish J Misra DP Yarandi H. Acculturation and postpartum depression among immigrant women of arabic descent. J Immigr Minor Health. (2019) 21(6):1208–16. 10.1007/s10903-018-0837-z 30456725

[31] Khalil D George Z Dannawey E Hijawi J ElFishawy S Jenuwine E. Maternal stressors and maternal bonding among immigrant and refugee Arab Americans resettled in the United States. Res Nurs Health. (2024) 47(2):141–50. 10.1002/nur.22365 38149856 PMC11440619

[32] Awaad R El-Gabalawy O Jackson-Shaheed E Zia B Keshavarzi H Mogahed D. Suicide attempts of Muslims compared with other religious groups in the US. JAMA Psychiatry. (2021) 78(9):1041–2. 10.1001/jamapsychiatry.2021.1813 34287614 PMC8295887

[33] Alhomaizi D Alsaidi S Moalie A Muradwij N Borba CP Lincoln AK. An exploration of the help-seeking behaviors of Arab-Muslims in the US: a socio-ecological approach. J Muslim Ment Health. (2018) 12(1):19–48. 10.3998/jmmh.10381607.0012.102

[34] Awaad R Ali S Elsayed D Zia B Elahi S. Development and validation of the Muslims’ perceptions and attitudes to mental health (M-PAMH) scale with a sample of American Muslim women. J Muslim Ment Health. (2019) 13(2):2. 10.3998/jmmh.10381607.0013.205

[35] Vu M Muhammad H Peek ME Padela AI. Muslim women’s perspectives on designing mosque-based women’s health interventions: an exploratory qualitative study. Health Educ Behav. (2017) 45(1):112–20. 10.1080/03630242.2017.1292344 28278014 PMC5634916

[36] Yeo S Kim-Hines Y Ehiri J Magrath P Johnson-Agbakwu C Ernst K. A qualitative study exploring the factors influencing maternal healthcare access and utilization among Muslim refugee women resettled in the United States. PLoS One. (2024) 19(8):e0307192. 10.1371/journal.pone.0307192 39150938 PMC11329121

[37] Abdel-Salam L Rifkin R Smith L Zaki S. Experiences of gender among Arab American women: a qualitative study. J Couns Psychol. (2019) 66(3):255–68. 10.1037/cou0000329 30714745

[38] Chaudhry A Hebert-Beirne J Alessi EJ Khuzam MZ Mitchell U Molina Y. Exploring the health impact of intersectional minority identity stressors on Arab sexual minority women migrants to the United States. Qual Health Res. (2025) 35(1-2):149–63. 10.1177/10497323241265288 39172019

[39] McLaughlin MM Ahmad SS Weisman de Mamani A. A mixed-methods approach to psychological help-seeking in Muslims: Islamophobia, self-stigma, and therapeutic preferences. J Consult Clin Psychol. (2022) 90(7):568–81. 10.1037/ccp0000746 35901369

[40] Rashed D Moreno G Nze C. Integration of Arab and Muslim health education into a medical school curriculum. MedEdPORTAL. (2022) 18:11188. 10.15766/mep_2374-8265.11188

[41] Khalil D Fry-McComish J Yarandi HN Giurgescu C. Arab Muslims’ perceptions of perinatal loss care in the United States. MCN Am J Matern Child Nurs. (2021) 47(1):30–7. 10.1097/NMC.0000000000000768

[42] Meyer IH. Prejudice, social stress, and mental health in lesbian, gay, and bisexual populations: conceptual issues and research evidence. Psychol Bull. (2003) 129(5):674–97. 10.1037/0033-2909.129.5.674 12956539 PMC2072932

[43] Crenshaw K. Demarginalizing the intersection of race and sex: a black feminist critique of antidiscrimination doctrine, feminist theory, and antiracist politics. Univ Chic Leg Forum. (1989) 1989(1):139–67.

[44] Crenshaw K. Mapping the margins: intersectionality, identity politics, and violence against women of color. Stanford Law Rev. (1991) 43(6):1241–99. 10.2307/1229039

[45] Cerda IH Macaranas AR Liu CH Chen JA. Strategies for naming and addressing structural racism in immigrant mental health. Am J Public Health. (2023) 113(S1):S38–44. 10.2105/AJPH.2022.307165 PMC9877373 36696610

[46] Pederson AB Waldron EM Fokuo JK. Perspectives of black immigrant women on mental health: the role of stigma. Womens Health Rep. (2022) 3(1):307–17. 10.1089/whr.2021.0071 PMC8994434 35415711

[47] Tanhan A Young JS. Muslims and mental health services: a concept map and a theoretical framework. J Relig Health. (2021) 60(6):3926–62. 10.1007/s10943-021-01324-4 PMC8267770 34241742

[48] Tervalon M Murray-García J. Cultural humility versus cultural competence: a critical distinction in defining physician training outcomes in multicultural education. J Health Care Poor Underserved. (1998) 9(2):117–25. 10.1353/hpu.2010.0233 10073197

[49] De las Nueces D Hacker K DiGirolamo A Hicks LS. A systematic review of community-based participatory research to enhance clinical trials in racial and ethnic minority groups. Health Serv Res. (2012) 47(3 Pt 2):1363–86. 10.1111/j.1475-6773.2012.01386.x 22353031 PMC3418827

[50] Wang P Hernandez R Fernandez ME Reininger B Wells R Crum M. Effectiveness of community-based participatory research (CBPR) interventions on mental health outcomes: a systematic review. Soc Sci Med. (2024) 363:117477. 10.1016/j.socscimed.2024.117477 39536652

[51] U.S. Office of Management and Budget. Revisions to OMB’s statistical policy directive No. 15: standards for maintaining, collecting, and presenting federal data on race and ethnicity. Fed Regist. (2024) 89(62):22182–216. Available online at: federalregister.gov [1, 2, 3].

