# Supplementary material for: Mental health, resilience, and culturally congruent care among Muslim and Arab American women in the United States: a scoping review
Source: Front Glob Womens Health. 2026 Oct 16;7:1931567. doi: 10.3389/fgwh.2026.1931567 (PMC13720362; doi:10.3389/fgwh.2026.1931567)
Supplement: Supplementary file 1 [file Supplementaryfile1.pdf]

Appendix A

Database search strategy and stringed keywords

| Database | Search String | Search Date | Filters Applied | # |
|---|---|---|---|---|
| **PubMed /MEDLINE** | (Arab*[tiab] OR Muslim*[tiab] OR "Middle East immigrants" [tiab] OR "Middle Eastern" [tiab] OR "Arabs" [MeSH Terms] OR "Muslims"[MeSH Terms])<br>AND<br>(woman[tiab] OR women[tiab] OR female*[tiab] OR "Women" [MeSH Terms])<br>AND<br>("mental health" [tiab] OR "mental disorder*" [tiab]<br>OR depress*[tiab] OR anxi*[tiab] OR distress[tiab]<br>OR wellbeing[tiab] OR "well-being"[tiab] OR resilience[tiab] OR coping[tiab] OR stigma[tiab]<br>OR discrimination[tiab] OR "help-seeking"[tiab]<br>OR "healthcare"[tiab] OR "health care"[tiab]<br>OR "health services"[tiab] OR "cultural competence"[tiab] OR "culturally competent"[tiab]<br>OR "cultural humility"[tiab])<br>AND<br>("United States"[MeSH Terms] OR "United States"[tiab] OR American*[tiab]) | 16 June 2026 | Humans; English language; Publication date 2016/01/01–2026/06/16; Journal article | 295 |
| **CINAHL (EBSCOhost)** | (MH "Arabs+" OR MH "Muslims+" OR TX Arab* OR TX Muslim* OR TX "Middle Eastern") AND (MH "Women's Health+" OR TX women OR TX female*) AND (MH "Mental Health+" OR MH "Mental Disorders+" OR TX "mental health" OR TX depression OR TX anxiety OR TX "psychological distress" OR TX "help-seeking" OR MH "Health Services Accessibility+") AND (MH "Culturally Competent Care+" OR TX "culturally congruent" OR TX "culturally competent" OR TX "cultural humility") AND (MH "United States+" OR TX "United States" OR TX US OR TX USA) | 16 June 2026 | Peer reviewed; English language; Publication date 20160101–20260616 | 319 |
| **Scopus** | TITLE-ABS-KEY(("Arab*" OR "Muslim*" OR "Middle Eastern") AND ("women" OR "female*") AND ("mental health" OR "depression" OR "anxiety" OR "psychological distress" OR "wellbeing" OR "well-being" OR "help-seeking" OR "help seeking") AND ("culturally congruent" OR "culturally competent" OR "cultural humility" OR "culturally responsive")) AND (LIMIT-TO(LANGUAGE, "English")) AND PUBYEAR > 2015 AND PUBYEAR < 2027 | 16 June 2026 | Document type: Article; Language: English; Subject area: Medicine, Nursing, Psychology, Social Sciences; Publication years 2016–2026 | 417 |
| Google Scholar | ("Arab American" OR "Muslim American") women mental health "culturally congruent" OR "culturally competent" United States | 16 June 2026 | First 200 results screened by relevance; English language; 2016–2026; no | 107 |

| | | | | |
|---|---|---|---|---|
| (supplementary/manual) | | | peer-review filter available (screened manually for peer-reviewed status) | |
