# Supplementary material for: Mental health, resilience, and culturally congruent care among Muslim and Arab American women in the United States: a scoping review
Source: Front Glob Womens Health. 2026 Oct 16;7:1931567. doi: 10.3389/fgwh.2026.1931567 (PMC13720362; doi:10.3389/fgwh.2026.1931567)
Supplement: Supplementary file 2 [file Supplementaryfile2.pdf]

Appendix B

Data abstraction table

Author(s) & Year	Study Design	Sample/ Population	U.S. Setting	Mental Health Concept(s)	Key Findings	Culturally Congruent Care Elements	Barriers Identified	Protective/Resilience Factors
**Almutairi et al. (2022)**	Scoping review (23 studies)	Middle Eastern immigrant women, U.S. (Including Muslim American women)	Multiple U.S. settings	Depression, anxiety, PTSD, MH service utilization	High prevalence of MH disorders; low utilization of MH services reported across studies; acculturative stress, discrimination, and language barriers were key factors	Calls for culturally tailored MH services and provider training	Language barriers, stigma, lack of culturally competent providers, limited access	Social support, community networks
**Simonovich et al. (2022)**	Mixed-methods scoping review (9 studies)	Pregnant and postpartum Muslim American women	Clinical and community perinatal settings	Perinatal anxiety and depression	Muslim faith practices (prayer, Quranic recitation, Dua, audio recordings of prayers) significantly reduce perinatal anxiety and depression symptoms	Faith-integrated perinatal care; recommends incorporating non-pharmacological faith practices in clinical settings	Stigma around perinatal MH in Muslim communities; underrepresentation in Western literature	Prayer, Quranic recitation, Dua, faith community support
**Berry (2025)**	Scoping review (31 studies)	Arab American women refugees in U.S. (and Canada)	U.S. and Canadian primary and community care settings	PTSD, depression, anxiety, barriers to care	MH challenges compounded by stigma, language barriers, and inadequate culturally sensitive care; both individual	Calls for culturally sensitive care models; community-level interventions recommended	Stigma, language, lack of culturally competent providers, structural access barriers	Community social support, religious coping

					and structural barriers identified			
**Abuelezam et al. (2018)**	Comprehensive literature review	Arab Americans, U.S., including women	U.S. healthcare broadly	Depression, psychological distress, MH disparities	Arab Americans face MH disparities linked to discrimination, immigration stress, and social marginalization; depression rates; MENA category exclusion from Census hampers research	Highlights need for culturally responsive care and targeted health policy	Discrimination, census invisibility, lack of MH data disaggregation, stigma	Family cohesion, religious identity
**Elshahat & Moffat (2022)**	Scoping review	Arabic-speaking immigrant and refugee women and men, North America (includes Arab American women)	Community and healthcare settings, U.S. and Canada	Depression, PTSD, anxiety, wellbeing	Pre-migration trauma, acculturative stress, discrimination, and language barriers are key risk factors; social support and community connections are protective; North American studies confirm elevated MH burden	Recommends culturally adapted interventions acknowledging both pre- and post-migration stressors	Pre-migration trauma, acculturation stress, language barriers, discrimination	Social support, community ties, religious coping
**Elahi et al. (2023)**	Cross-sectional quantitative	Muslim American women, n=1,214, U.S.	Community recruitment across U.S.	MH beliefs, help-seeking attitudes, rejection of formal MH services	Significant ethnic differences in MH beliefs and rejection attitudes; Arab Muslim women show lower	Underscores need for ethnicity-specific outreach and culturally tailored MH	Stigma, cultural beliefs about MH causation, low familiarity with services	Not examined

					familiarity with professional MH services than White Muslims; ethnicity moderated beliefs about MH causation and service use	literacy programs		
**Ali et al. (2022)**	Cross-sectional quantitative	Muslim women in the U.S.	Community, U.S. (Stanford lab sample)	Rejection attitudes toward formal MH services	Religiosity, beliefs about MH, and perceived stigma predicted rejection of formal MH services; Muslim women significantly underutilize professional MH care; cultural and religious norms shaped attitudes	Recommends faith-concordant MH messaging and provider cultural humility training	Stigma, religious beliefs about MH, cultural norms, distrust of Western therapeutic models	Religiosity as potential bridge if destigmatization occurs
**McLaughlin et al. (2022)**	Mixed-methods (survey + qualitative)	Muslims in the U.S. including women, community sample	Community recruitment, U.S.	Help-seeking, Islamophobia, self-stigma	Perceived Islamophobia positively associated with self-stigma of seeking help, which further predicted negative help-seeking attitudes. Participants preferred same-religion or culturally competent	Therapist demographic and treatment modality preferences highlighted; need for Muslim-affirming therapy	Islamophobia, self-stigma, preference for same-faith or culturally competent providers, lack of available Muslim therapists	Community belonging, faith

					therapists; Muslim women reported stronger stigma			
**Baltimore & Kim (2024)**	Narrative review	Middle Eastern migrant women, U.S. (Including Muslim Americans, Arab Americans, and Muslim Arab Americans)	Social work and community health settings	MH service utilization, MH literacy	Low MH literacy, stigma, and cultural barriers drive underutilization among Middle Eastern migrant women; social workers play a critical role in bridging access gaps through community-based outreach	Social work interventions; MH literacy promotion; culturally responsive practice frameworks	MH stigma, language barriers, cultural norms, distrust of formal systems	Social work relationship-building, community trust
**Alhomaizi et al. (2018)**	Qualitative (socio-ecological)	Arab-Muslim individuals including women, U.S. (includes Arab American, Arab Muslim and Muslim American women)	Community and MH settings, U.S.	Help-seeking behaviors	Identified multi-level barriers across individual (stigma, shame), community (family and community pressure), and structural (provider bias, lack of Muslim providers) levels; women faced additional gendered barriers around shame and family honor	Socio-ecological framework; recommends community-level and provider-level interventions	Shame, family/community pressure, lack of Muslim MH providers, provider bias, distrust	Mosque community, family support when destigmatized
**Murrar et al. (2024)**	Cross-sectional quantitative	Muslim American women	Medical settings, outpatient and	Perceived discrimination in medical settings	25.4% reported being treated with less courtesy than	Highlights need for implicit bias training and culturally	Provider discrimination, religious bias,	Religiosity as potential buffer

		(n=254), Chicago, IL	community, Chicago		non-Muslims; 29.8% reported providers did not listen to them; 24.3% reported providers acted as if they were not smart; higher self-rated religiosity was negatively associated with discrimination	competent provider education	hijab-related stereotyping	
**Hashem & Awad (2024)**	Cross-sectional quantitative	Muslim American women (n=123), U.S.	Community settings, U.S.	Discrimination, psychological distress (anxiety, somatization, paranoia)	Solo status (being the only Muslim in a setting) moderated the relationship between discrimination and distress; effect differed for hijab-wearing vs. non-hijab-wearing women; solo status amplified distress particularly for consistent hijab wearers	Recommends reducing Muslim women's solo status in healthcare and professional settings through inclusive hiring and representation	Islamophobia, discrimination, hijab-related stereotyping, social isolation	Community presence, same-faith peers
**Budhwani & Hearld (2017)**	Cross-sectional quantitative (online survey)	Muslim American women (n=373), U.S.	U.S. community (online)	Depression, internalized stigma, physical and sexual abuse	Internalized stigma (heightened vigilance) significantly associated with depression	Calls for stigma-reducing clinical interventions sensitive to Muslim identity	Internalized stigma, abuse, discrimination, low income	Not examined

					(OR=0.924, 95% CI=0.888–0.962, p<0.001); physical abuse (30.3%) and sexual abuse (15.5%) prevalent; lower income and less education also predicted depression			
**Abuelezam & Fontenot (2017)**	Narrative review	Arab American and Arab immigrant women, U.S.	U.S. outpatient and community health settings	Depression, IPV, social support	Elevated rates of depression among Arab American women linked to immigration stress, IPV, isolation, acculturation conflict, and cultural gender role expectations; limited culturally competent depression screening tools available	Recommends culturally adapted depression screening and provider training	Language barriers, IPV, acculturation stress, lack of culturally adapted screening tools	Family support, religious faith
**Saherwala et al. (2021)**	Clinical case-based review	Muslim American women in the U.S. (case illustrations, Wright State)	Outpatient psychiatric/MH settings, Ohio	Depression, PPD, stigma, cultural beliefs about MH	Case-based analysis demonstrates that postpartum depression, medication stigma, modesty concerns, discrimination	Provides practical framework: acknowledge Islamic beliefs, avoid invalidating religious coping, address	Stigma against MH medications, modesty concerns, cultural beliefs about mental illness causation, provider unfamiliarity with Islam	Religiosity, prayer, family support

					effects, and unique cultural beliefs are core challenges; cultural competence in therapeutic alliance is essential	medication stigma, respect modesty; recommends female providers where possible		
**Vu et al. (2016)**	Cross-sectional quantitative survey	Muslim American women (n=254), Chicago, IL	Mosque and community events, Chicago	Delayed healthcare seeking	53% reported delays in care due to perceived lack of female clinicians; higher religiosity (OR=5.2, $p<0.01$) and higher modesty (OR=1.4, $p<0.001$) positively predicted delayed care; residing in U.S. >20 years negatively associated with delay (OR=0.22, $p<0.05$)	Recommends healthcare systems increase availability of female providers; advocates for religion-sensitive care accommodations	Lack of female clinicians, modesty concerns, high religiosity, fatalistic beliefs	Longer U.S. residency as protective factor against care delay
**Rashed et al. (2022)**	Curriculum development/educational study	Medical students and healthcare providers, U.S. (Subject of focus: Arab American patients and	Medical school, U.S.	Provider MH literacy, culturally competent care training	Development and implementation of a curriculum module integrating Arab and Muslim health into medical education;	Direct training intervention for providers: Islamic dietary practices, gender dynamics, mental health stigma, end-of-life beliefs	Provider knowledge gaps, absence of Arab/Muslim content in standard medical curricula	Not applicable (provider-focused)

		Muslim American patients			pre/post assessment showed improved provider knowledge of cultural and religious health needs	integrated into medical curriculum		
**Vu et al. (2017)**	Qualitative exploratory study (n=19)	Muslim American women, Chicago, IL	Mosque-based setting, Chicago	Women's health intervention design, culturally congruent care preferences	Women preferred mosque-based interventions; wanted female-only spaces; emphasized modesty, privacy, and Islamic values in intervention design; spiritual leaders viewed as trusted messengers	Mosque-based delivery; female-only programming; integration of Islamic values; community health workers; imams as collaborators	Distrust of formal healthcare, modesty, preference for gender-concordant and faith-aligned settings	Faith, mosque community, peer networks
**Awaad et al. (2022)**	Community-based participatory study	Muslim community members including women, San Francisco Bay Area (including Muslim American women)	Community mental health, mosque-based, Bay Area, CA	Community MH partnerships, psychoeducation	Mosque-based mental health community advisory board model demonstrated feasibility and acceptability; participants reported increased MH literacy and reduced stigma; family-centered, faith-integrated approach was	Community-based participatory research model; mosque partnerships; faith-integrated psychoeducation; family engagement	Stigma, distrust of clinical MH services, language, fear of community judgment	Mosque community, faith leaders, family involvement

					key to engagement			
**Alhasanat-Khalil et al. (2018)**	Cross-sectional quantitative (n=115)	Immigrant Arab American women, 1–12 months postpartum, Dearborn.	Clinics in Dearborn, Michigan (Arab community center)	PPD, acculturative stress, social support	Higher acculturative stress, higher education, antenatal anxiety, and lower social support all significantly predicted PPD symptoms ($p<0.05$); 88% had annual household income <$40,000; 80% unemployed; 68% preferred Arabic interview	Recommends culturally adapted PPD screening in Arabic; antenatal anxiety screening for Arab immigrant women	Acculturative stress, language, low income, unemployment, lack of social support	Not examined
**Alhasanat-Khalil et al. (2019)**	Cross-sectional quantitative (n=115)	Immigrant Arab American women, 1–12 months postpartum, Ohio and Michigan	University hospital outpatient clinics, Ohio and Michigan	PPD, acculturation, marginalization	25% of women (n=29) scored ≥10 on EPDS (indicating PPD symptoms); marginalization was positively associated with more PPD symptoms ($r=.25$, $p=.008$); acculturation (attraction to Arabic or American culture) not significant after adjustment	Recommends antenatal anxiety screening and culturally adapted PPD assessment tools for Arab immigrant women	Marginalization, acculturation conflict, language barriers, low social support	Arabic cultural identity, family support

**Khalil et al. (2024)**	Cross-sectional quantitative	Immigrant and refugee Arab American women, Wayne State, Detroit, MI	Community centers and clinics, Detroit, MI	Maternal stress, trauma, depressive symptoms, maternal bonding	Acculturative stress, trauma exposure, and depressive symptoms significantly predicted impaired maternal bonding; findings first to document this relationship in Arab American immigrant/refugee mothers	Calls for integrated trauma-informed and culturally congruent perinatal care; screening for acculturative stress and depressive symptoms	Trauma, acculturative stress, depression, isolation	Not examined
**Yeo et al. (2024)**	Qualitative (in-depth interviews, n=17)	Afghan, Iraqi, and Syrian Muslim refugee women, Arizona (Muslim Americans and Muslim Arab Americans)	Maternal healthcare clinics and community settings, Arizona	Maternal healthcare access, cultural humility, language	Micro-level factors: women's attitudes toward hospitals and prenatal care, language proficiency; meso-level: provider cultural competence, interpreter availability; macro-level: immigration policy, healthcare system navigation; language and fear of system were predominant barriers	Cultural humility from providers; professional interpreters; community health workers; patient navigation; family-inclusive care	Language barriers, lack of cultural humility from providers, immigration fears, lack of interpreters	Community networks, family involvement, faith

**Khalil et al. (2021)**	Cross-sectional survey	Arab Muslim American women experiencing perinatal loss, U.S.	Hospital-based perinatal settings, U.S.	Perinatal loss, bereavement, culturally congruent care	Arab Muslim women reported unmet spiritual and cultural needs during perinatal loss care; providers lacked knowledge of Islamic bereavement practices (e.g., washing the body, burial timing, prayers); women felt culturally unsupported	Recommends Islamic bereavement protocols in perinatal units; education for nurses on Islamic funeral practices and religious coping with loss	Provider unfamiliarity with Islamic practices, absence of faith-informed bereavement protocols	Islamic faith, prayer, religious community support
**Norris & Aroian (2016)**	Descriptive cross-sectional quantitative	Severely traumatized Arab immigrant women, U.S. (Arab American women)	Community mental health settings, U.S.	PTSD, MDD, pre/post-migration stressors	Pre-migration persecution significantly predicted PTSD; post-migration stressors (discrimination, acculturative stress) also contributed; social support was the strongest protective post-migration resource; high rates of both PTSD and MDD documented	Recommends trauma-informed, culturally adapted MH interventions addressing both pre- and post-migration stressors	Pre-migration persecution, post-migration discrimination, acculturative stress, lack of social support	Social support as key protective factor post-migration
**Chaudhry et al. (2025)**	Qualitative (semi-structured interviews, n=20)	Arab sexual minority women	Community and MH settings, U.S.	Intersectional minority stress, MH impact	Participants experienced compounded	Recommends intersectionality-informed,	Triple minority stress, fear of family and	LGBTQ+ community support, chosen family,

		migrants, U.S.			stressors from Arab ethnic identity, Muslim religious identity, and sexual minority identity simultaneously; triple minority stress significantly impacted mental health; fear of family rejection and community ostracism were prominent	affirming care that addresses ethnic, religious, and sexual minority identities together	community rejection, lack of affirming providers, immigration precarity	anonymity of U.S. urban settings
**Abdel-Salam et al. (2019)**	Qualitative (CQR, n=11)	Arab American women, U.S.	Counseling psychology context, U.S.	Gender, ethnic identity, psychological wellbeing	Identified themes: experiences of gendered ethnic expectations (modesty, marriage pressure, family honor), navigating between Arab and American cultural norms, discrimination based on both ethnicity and gender, and psychological strain from dual identity management	Recommends culturally competent counseling acknowledging intersections of gender and Arab ethnicity; culturally adapted therapeutic approaches	Dual cultural identity conflict, gendered ethnic expectations, discrimination, limited access to culturally knowledgeable counselors	Bicultural identity integration, family support
**Awaad et al. (2019)**	Scale development and validation	Muslim American	Community, U.S.	MH perceptions,	Developed and validated the M-	Provides validated	Negative MH beliefs, rejection	Not examined

		women, U.S. (Stanford MMHIP sample)		attitudes, help-seeking	PAMH scale with Muslim women; identified three key factors predicting MH service underutilization: negative beliefs about MH, rejection attitudes toward services, and low familiarity with MH care; scale showed good internal consistency and validity	assessment tool for measuring Muslim women's MH attitudes; enables targeted destigmatization interventions	attitudes, low MH literacy	
**Awaad et al. (2021)**	Cross-sectional nationally representative survey (ISPU 2019 poll)	Muslim Americans, U.S. national sample (men and women)	National community-based survey, U.S.	Suicidal ideation and suicide attempts	Muslim Americans had twice the rate of lifetime suicide attempts compared to all other religious groups (8% vs. 6% Catholic, 5% Protestant, 3.6% Jewish); religiosity level did not affect odds; discrimination and stigma identified as key drivers	Urgent call for culturally and religiously congruent suicide prevention and postvention programs in Muslim communities	Mental health stigma, religious prohibition on discussing suicide, discrimination, lack of Muslim MH providers	Imam-led community outreach proposed as culturally appropriate intervention channel
